# Association Between State Medicaid Expansion and Emergency Access to Acute Care Hospitals in the United States

**DOI:** 10.1001/jamanetworkopen.2020.25815

**Published:** 2020-11-16

**Authors:** David J. Wallace, Julie M. Donohue, Derek C. Angus, Lindsay M. Sabik, Billie Davis, Jonathan Yabes, Jeremy M. Kahn

**Affiliations:** 1 Department of Critical Care Medicine, University of Pittsburgh School of Medicine, Pittsburgh, Pennsylvania; 2 Department of Emergency Medicine, University of Pittsburgh School of Medicine, Pittsburgh, Pennsylvania; 3 Department of Health Policy and Management, University of Pittsburgh Graduate School of Public Health, Pittsburgh, Pennsylvania; 4 Department of General Internal Medicine, University of Pittsburgh School of Medicine, Pittsburgh, Pennsylvania

## Abstract

**Importance:**

State decisions not to expand Medicaid under the Patient Protection and Affordable Care Act could reduce emergency access to acute care hospitals.

**Objective:**

To determine the relationship between state Medicaid expansion and emergency access to acute care hospitals in the United States.

**Design, Setting, and Participants:**

This cross-sectional study linked hospital-level data from the Centers for Medicare & Medicaid Services from 2007 to 2017 to US Census data for all 50 US states and the District of Columbia. Geospatial analyses and difference-in-differences regression models were used to compare temporal changes in the size of the population without 30-minute access to acute care hospitals between 32 states that expanded Medicaid with the population without access in 19 that did not, before and after expansion. Analyses focused on the total population and those with low incomes; secondary analyses examined emergency access to safety-net hospitals.

**Exposures:**

State-level Medicaid expansion.

**Main Outcomes and Measures:**

Population without emergency access to an acute care hospital, defined as living outside a 30-minute drive of any hospital.

**Results:**

States that did not expand Medicaid experienced an increase in the population without access to hospitals overall (without expansion: 6.76% to 6.79% [0.03%]; vs with expansion: 5.65% to 5.35% [–0.30%]; difference-in-differences, 0.33%; 95% CI, 0.33%-0.34%; *P* < .001) and for low-income persons (without expansion: 7.43% to 7.39% [–0.04%]; vs with expansion: 6.25% to 6.15% [–0.10%]; difference-in-differences, 0.06%; 95% CI, 0.05%-0.07%; *P* < .001). If access changes in nonexpansion states were the same as expansion states, an estimated 421 000 more persons overall and 48 000 more low-income persons would have retained access. States that did not expand Medicaid experienced an increase in the population without access to safety-net hospitals overall (46.91% to 47.70% [0.79%] vs 33.94% to 33.07% [–0.87%]; difference-in-differences, 1.66%; 95% CI, 1.64%-1.66%; *P* < .001) and for low-income persons (45.28% to 46.14% [0.86%] vs 33.00% to 32.23% [–0.77%]; difference-in-differences, 1.63%; 95% CI, 1.63%-1.67%; *P* < .001). If access changes in nonexpansion states were the same as expansion states, an estimated 2 242 000 more persons overall and 364 000 more low-income persons would have retained access.

**Conclusions and Relevance:**

States that did not expand Medicaid under the Patient Protection and Affordable Care Act were associated with worse emergency access to acute care hospitals compared with states that expanded Medicaid.

## Key Points

**Question:**

Are states that chose not to expand Medicaid under the Patient Protection and Affordable Care Act associated with reduced emergency access to acute care hospitals?

**Findings:**

In this cross-sectional study of acute care hospital availability in all 50 US states and the District of Columbia, states that did not expand Medicaid experienced worsened emergency access to acute care hospitals compared with states that expanded Medicaid.

**Meaning:**

This study found reduced emergency access to acute care hospitals in states that did not expand Medicare, which could negatively impact the quality of care for time-sensitive conditions such as acute myocardial infarction, stroke, sepsis, and trauma.

## Teaser

This cross-sectional study uses hospital-level Medicaid and geospatial data to analyze the association of state expansion of Medicaid under the Patient Protection and Affordable Care Act with emergency access to acute care hospitals.

## Introduction

Timely access to acute care services can be lifesaving in medical emergencies such as acute myocardial infarction,^1,2,3^ stroke,^4,5,6^ sepsis,^7,8,9^ and trauma.^10,11,12,13^ For decades, the primary approach to maintain or improve access to acute care hospitals for patients with medical emergencies has been to implement organizational approaches such as regionalization of acute care.^14,15,16^ In contrast, relatively little attention has been paid to the role of insurance reform, sources of hospital revenue, and the financial sustainability of hospitals providing emergency services. Signed into law in 2010, the Patient Protection and Affordable Care Act (ACA) included provisions for states to receive enhanced matching federal funds to expand eligibility for Medicaid up to 138% of the federal poverty level for adults. As of December 2017, 19 states had not expanded coverage to those newly eligible under the ACA. Evidence suggests that the decision not to adopt Medicaid expansion has contributed to hospital closures in those states.^17^ However, the extent to which hospital closures have affected access to care is not known. As hospital closures could occur in areas with duplication of services or in areas with declining populations, fewer hospitals does not necessarily translate to decreased population access. At the same time, closures of safety-net hospitals specifically may constitute a practical loss of access for some patients, even if other nearby hospitals remain open, as underinsured persons may be dissuaded from accessing services because of the potential for high out-of-pocket expenses.

To address this knowledge gap, we evaluated the association of Medicaid expansion under the ACA with changes in emergency access to acute care hospitals in the overall and low-income US population. We examined access both to short-term acute care hospitals overall and to safety-net hospitals, as safety-net hospitals are potentially more sensitive to changes in uncompensated care.^18,19,20,21^ We evaluated both overall and low-income population access, as the low-income population was specifically targeted for coverage expansions under the proposed Medicaid eligibility changes.

## Methods

Our analyses involved 3 linked steps: (1) identifying and geolocating all short-term acute care hospitals in the United States; (2) estimating populations without emergency access to acute care hospitals, which we defined as living outside a 30-minute driving distance of any hospital, and (3) using a difference-in-differences approach to compare changes in population access to acute care hospitals in states that expanded Medicaid with those that did not. The study included all 50 US states and the District of Columbia. As all analyses used aggregated population and hospital-level data, the project did not meet criteria for human subjects research and informed consent requirements according to the University of Pittsburgh Human Research Protection Office. We adhered to the Strengthening the Reporting of Observational Studies in Epidemiology (STROBE) reporting guideline for cross-sectional studies.

### Data Sources

We used 5 data sources. First, to identify short-term acute care hospitals we used the Centers for Medicare & Medicaid Services (CMS) annual Healthcare Cost Report Information System (HCRIS) data for the years 2007 through 2017.^22^ HCRIS reports, which are publicly available and produced by all facilities that receive CMS payments, include street addresses and select hospital characteristics. Second, to define safety-net hospitals we used publicly available CMS Supplemental Security Income files that report hospital-level services. Third, to estimate state-level annual populations we used US Census Bureau estimates aggregated from the zip code level for the years 2008 through 2017. The US Census Bureau produces annual population estimates based on a series of monthly samples, decennial population counts, and population changes based on immigration, emigration, births, and deaths.^23^ Fourth, to estimate the population of low-income individuals, we used the US Census Bureau’s American Community Survey 5-year samples to determine the population earning less than the federal poverty line in the prior 12 months, localized to the zip code level. As American Community Survey population poverty results are not available for the years 2008 through 2010, we created zip code–level estimates for those years based on linear growth population trajectories and absolute counts from 2011 through 2017. Fifth, for geolocation and rural location classification we used topologically integrated geographic encoding and referencing cartographic files from the US Census Bureau.^24^ To estimate driving times we used a 2012 Environmental Systems Research Institute road atlas and applied standard driving regulations.^25^

### Hospital Geolocations and Operational Status

We identified hospitals directly from HCRIS. We geolocated each hospital using their reported street address and categorized each hospital in each year either as new, existing, or closed relative to the hospital’s reporting in the prior and subsequent years. Hospitals that changed facility status from short-term acute care to any other facility type (eg, rehabilitation hospital) were considered closed, as under that change they would no longer provide essential services for time-sensitive medical emergencies. Hospitals that changed ownership but remained open in the same location without a gap in services were considered to be continuously open. Safety-net hospitals were defined as those in the highest quartile of hospitals in 2008 according to their percentage of patients eligible for Supplemental Security Income^26^ at the state level, as measured in HCRIS.^27^

We manually verified the operational status of all annual changes using a variety of methods, including Joint Commission records^28^ and internet searches. For hospitals that did not open, close, change location, or change ownership, we manually verified the operational status of a 5% random sample of hospitals using the same process. The final cohort included the years 2008 to 2017, with the year 2007 only used to assess hospital status in 2008.

As a point map of the United States showing new, closed, and unchanged hospital locations over the 10 years of study would be difficult to interpret because of areas with high hospital density and overlying locations, we transformed the US land area into a simplified uniform grid of 500–square mile hexagons, summarizing hospital changes that occurred within each hexagon during the entire study period. We chose 500–square mile hexagons as the shape has an inner circle diameter of 24 miles, approximating a 30-minute drive time catchment. We performed cartographic transformations in ArcGIS Pro 2.4 (ESRI). We created this map solely for visualization to show where acute care resources are changing in the US over time.

### State Medicaid Expansion Definition

We defined our primary exposure, state Medicaid expansion status, as either expansion or nonexpansion using the first complete calendar year of expansion in each state. We determined the status and timing of state decisions to adopt ACA Medicaid expansion between January 2014 and December 2017 using public reporting.^29^

### Measurement of Population Access to Hospital-Based Emergency Services

We defined the population without emergency access to an acute care hospital as the count and percentage of the state population that lived outside a 30-minute drive of any short-term acute care hospital. A 30-minute threshold was used because medical care delivered within this time frame is associated with improved outcomes for many emergencies through early stabilization and initiation of definitive care.^1,5,12,13^ Calculating drive times from home zip codes is appropriate given that a majority of acute myocardial infarctions,^30^ traumas,^31^ strokes,^32^ and general medical emergencies^33^ occur near or at home. We performed access calculations using denominators of all and low-income state residents (defined as those reporting incomes below the federal poverty line). We included all ages for both the numerator and denominator because short-term acute care hospitals are frequently the first point of hospital contact for time-sensitive emergencies for both adult and pediatric patients. We converted zip code regions into geometric centroids, using the center of each zip code to measure population access, and performed calculations using Network Analyst in ArcGIS 10.6 software. For safety-net hospital population access calculations we performed the same steps using the subset of safety-net hospitals.

### Statistical Analysis

To determine the association between Medicaid expansion and population access we used a difference-in-differences approach with state-year as the unit of analysis. We fit a series of linear regression models using cluster-robust variances. We fit a total of 4 models with 4 different dependent variables defined at the state-year level: total population access to any acute care hospital, low-income population access to any acute care hospital, total population access to a safety-net hospital, and low-income population access to a safety-net hospital. Each model included a relative term for year (with time zero being the year before Medicaid expansion in states that expanded eligibility and 2013 in states that did not), a term for Medicaid expansion status, an interaction term for relative year and Medicaid expansion status, and a state-year level error term. Models accounted for stable state-level characteristics before and after the year 2013 (or year of Medicaid expansion, for states that expanded later). This approach allows us to control for other factors that could be associated with changing population access by evaluating changes within the same state relative to the year of Medicaid expansion for that state.

To better understand the population impacted by changes in emergency acute care hospital access, we further estimated the change in access in 2017 that was potentially associated with Medicaid nonexpansion. We did this by projecting the differential change in population access from Medicaid expansion states onto nonexpansion states’ populations for both the total and the low-income populations of those states. These estimates show the projected changes in population access in nonexpansion states that might have occurred had Medicaid been universally expanded in 2014. We rounded population estimates to the nearest thousand to avoid the appearance of false precision.

We used Stata version 15.0 (StataCorp) for all difference-in-differences analyses. We applied a *P* value of <0.05 for all statistical 2-tailed tests. Additional analytic details are provided in eMethods and eTables 4, 5, and 6 of the Supplement.

## Results

### Population and Hospital Distribution Characteristics in 2008

In total, there were 4601 hospitals, including 1118 safety-net hospitals, serving 291.9 million Americans, of whom 41.7 million (14.3%) had low income. (Hospital and population characteristics of the United States in 2008 prior to the enactment of the ACA, both overall and by subsequent state-level Medicaid expansion status, are available in eTable 1 in the Supplement.) In 2008, there were 17.6 million (6.0%) persons who lived more than 30 minutes from the nearest acute care hospital (Medicaid expansion states, 10.1 million [5.6%] persons; nonexpansion states, 7.5 million [6.8%] persons), of whom 2.8 million (16.0%) had low income, and 112.2 million (38.4%) persons who lived more than 30 minutes from the nearest safety-net hospital (expansion states, 60.6 million [33.4%] people; nonexpansion states, 51.5 million [46.7%] persons), of whom 15.7 million (14.0%) had low income.

### Population and Hospital Distribution Characteristics in 2017

In 2017, 32 states had expanded Medicaid under the ACA and 19 had not (eTable 2 in the Supplement lists hospital and population characteristics). There were 4528 hospitals, including 1045 safety-net hospitals, serving 313.0 million Americans, of whom 45.6 million (14.6%) had low incomes. Overall hospital and safety-net hospital counts decreased both in states that expanded Medicaid and states that did not, with larger absolute closure counts for both in states that did not expand Medicaid (eTables 1 and 2 in the Supplement). The total, low-income, and rural populations of the United States saw an absolute increase between 2008 and 2017, though as a percentage of the total population, the rural population decreased by 4.1%, from 63.7 million (21.8%) to 65.6 million (21.0%) of the total United States population.

### Overall Hospital Closures in States That Did and Did Not Expand Medicaid

**Figure 1.**
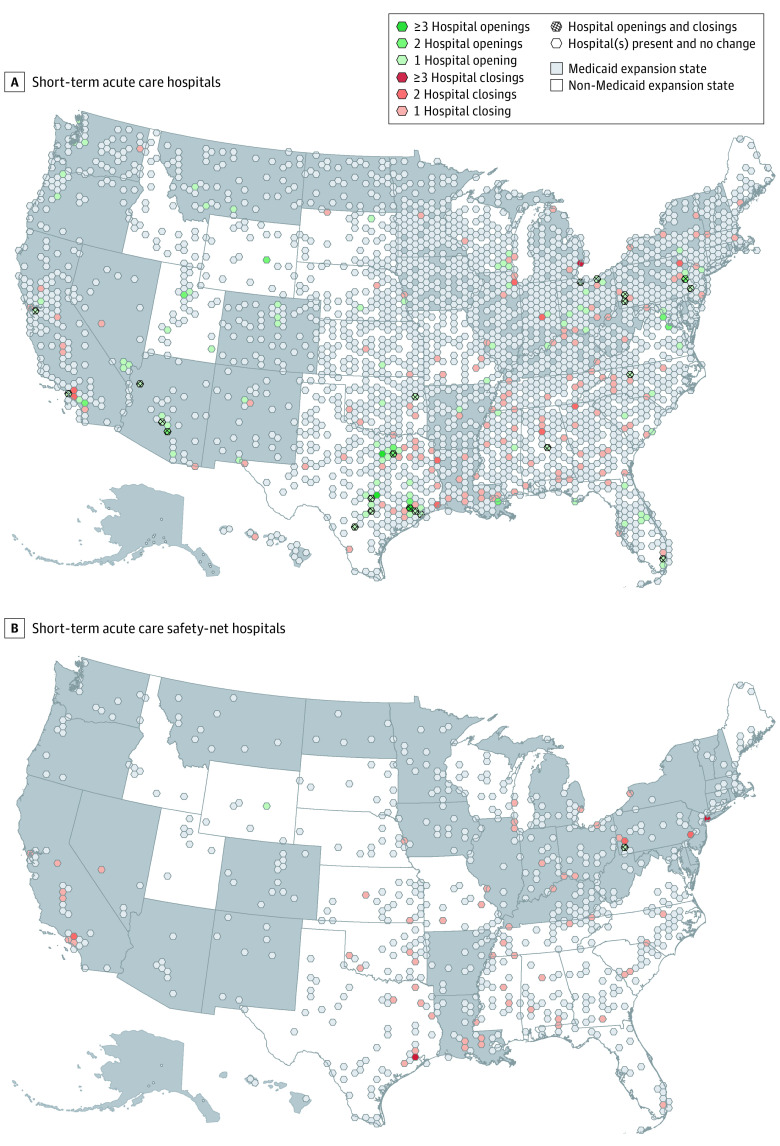
Change in Short-term Acute Care Hospitals and Short-term Acute Care Safety-Net Hospitals in the US by Medicaid Expansion Status Under the Patient Protection and Affordable Care Act The maps use binned hexagon tessellation to produce a visually interpretable compression of data at a national scale over the entire study interval. Each hexagon shows 500 square miles, with green areas showing areas with new hospital openings, red areas showing regions with permanent hospital closings, and semitransparent white hexagons showing regions without any hospital openings or closings. Hashed hexagons show regions with both openings and closings. States shaded gray expanded Medicaid under the Patient Protection and Affordable Care Act between 2014 and 2017. The map uses an equal area Albers projection.

**Figure 2.**
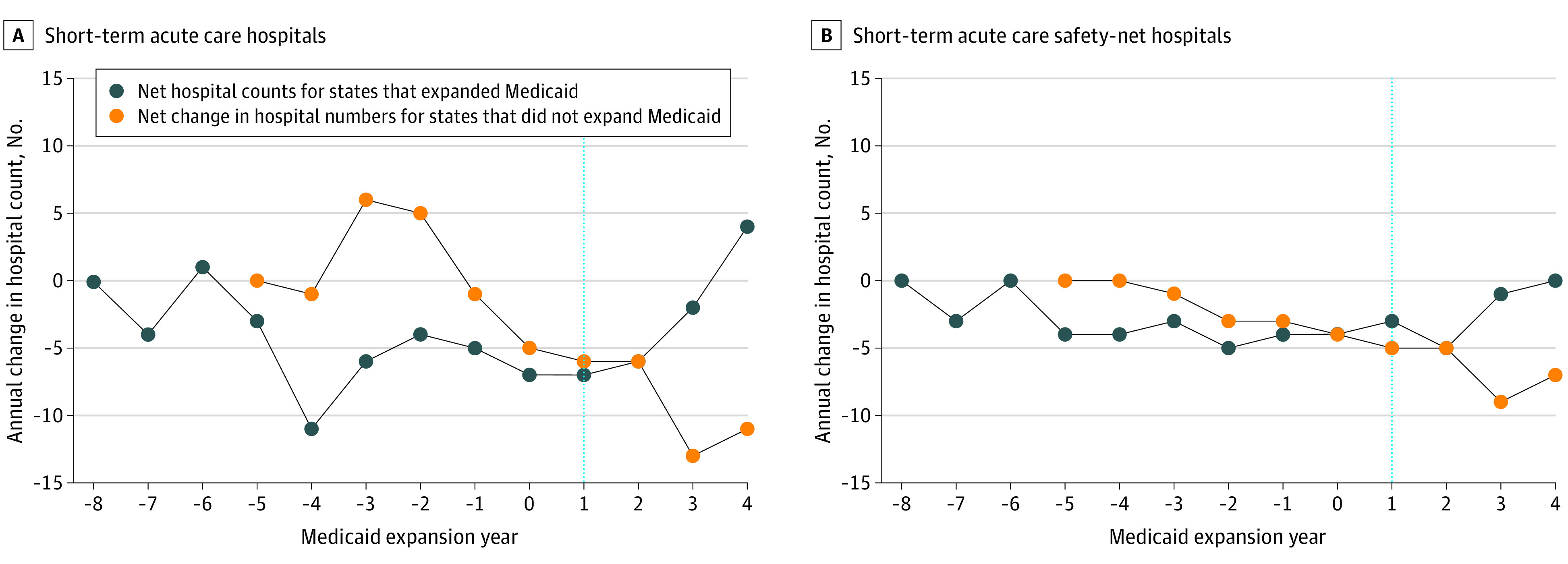
Annual Change in Short-term Acute Care Hospitals and Short-term Acute Care Safety-Net Hospitals in the US by Medicaid Expansion Status Under the Patient Protection and Affordable Care Act Thirty-two states expanded Medicaid eligibility requirements between 2014 and 2017 and 19 states did not. The blue dashed line indicates the first year of Medicaid expansion among states that expanded Medicaid and the year 2014 for states that did not.

There were 4735 total short-term acute care hospitals included in the longitudinal analysis of population access between 2008 and 2017. A total of 207 hospitals (4.4%) permanently closed or changed treatment designation and 125 hospitals (2.6%) newly opened, for a net reduction of 82 hospitals overall. Hospital closures occurred in most states (36 states [70.6%]) and with greatest frequency in the southeast United States (Figure 1). Both expansion and nonexpansion states experienced net hospital closures in most years (Figure 2).

### Difference-in-Differences Analyses of Population Access to All Hospitals

**Figure 3.**
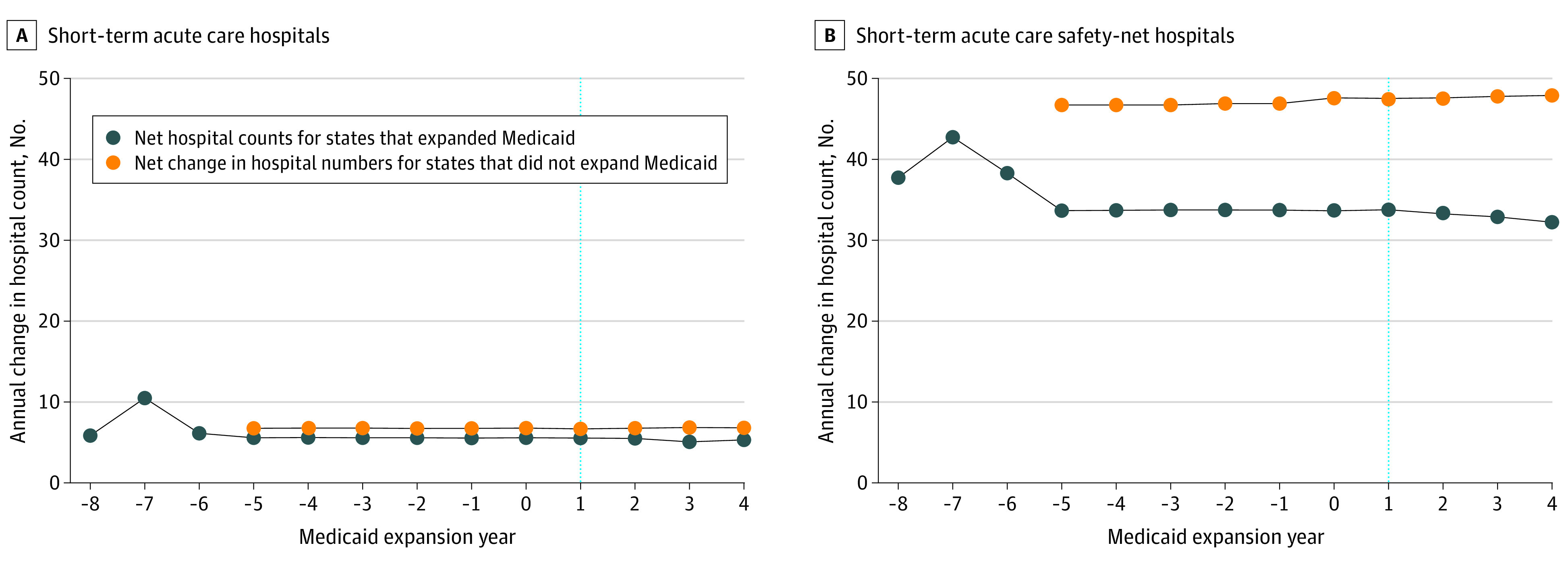
Percentage of Population Without Emergency Access to Any Short-term Acute Care Hospital or Short-term Acute Care Safety-Net Hospital by Medicaid Expansion Status Under the Patient Protection and Affordable Care Act Thirty-two states expanded Medicaid eligibility requirements between 2014 and 2017 and 19 states did not. The blue dashed line indicates the first year of Medicaid expansion among states that expanded Medicaid and the year 2014 for states that did not.

In the difference-in-differences analysis, states that did not expand Medicaid experienced an increase in the population without emergency access to an acute care hospital (6.76% to 6.79% [0.03%]) compared with states that expanded Medicaid (5.65% to 5.35% [–0.30%]), for a difference-in-differences of 0.33% (95% CI, 0.33% to 0.34%; *P* < .001; Table 1; Figure 3; eTable 3 in the Supplement). States that did not expand Medicaid experienced a decrease in the lower income population without emergency access to an acute care hospital (7.43% to 7.39% [–0.04%]) compared with states that expanded Medicaid (6.25% to 6.15% [–0.10%]), for a difference-in-differences of 0.06% (95% CI, 0.05%-0.07%; *P* < .001; Table 1; eTable 3 and eFigure 1 in the Supplement). The projected population impact of not expanding Medicaid was a loss of emergency access to the nearest hospital for 421 000 total persons and 48 000 lower-income persons in 2017 for states that did not expand Medicaid (Table 2). These counts represent 56.1% and 35.3% declines in population access to hospitals in nonexpansion states attributable to Medicaid nonexpansion.

**Table 1.** Population Without Access to Overall and Safety-Net Hospitals Before and After Medicaid Expansion^a^

Characteristics	Population, %		Difference	Difference-in-Differences (95% CI)	*P* value
	Before Medicaid expansion^b^	After Medicaid expansion^b^			
**No short-term acute care hospital <30-min drive for overall population**					
Expansion	5.65	5.35	–0.30	0.33 (0.33-0.34)	<.001
Nonexpansion	6.76	6.79	0.03	NA	NA
**No short-term acute care hospital <30-min drive for low-income population**					
Expansion	6.25	6.15	–0.10	0.06 (0.05-0.07)	<.001
Nonexpansion	7.43	7.39	–0.04	NA	NA
**No safety-net hospital <30-min drive for overall population**					
Expansion	33.94	33.07	–0.87	1.66 (1.64-1.66)	<.001
Nonexpansion	46.91	47.70	0.79	NA	NA
**No safety-net hospital <30-min drive for low-income population**					
Expansion	33.00	32.23	–0.77	1.63 (1.61-1.67)	<.001
Nonexpansion	45.28	46.14	0.86	NA	NA

Abbreviation: NA, not applicable.

^a^ Access data obtained from Centers for Medicare & Medicaid Services Healthcare Cost Report Information System annual reports and Supplemental Security Income files.^22,26^ State data obtained from public reporting as of December 2017. Expansion states were Alaska, Arkansas, Arizona, California, Colorado, Connecticut, District of Columbia, Hawaii, Iowa, Illinois, Indiana, Kentucky, Louisiana, Massachusetts, Maryland, Michigan, Minnesota, Montana, North Dakota, New Hampshire, New Jersey, New Mexico, Nevada, New York, Ohio, Oregon, Pennsylvania, Rhode Island, Vermont, Washington, and West Virginia. All other states were defined as nonexpansion.

^b^ Defined by the first full calendar year of Medicaid expansion in expansion states and 2014 in nonexpansion states.

**Table 2.** Estimated Change in Hospital Access Following Medicaid Expansion and Universal Medicaid Expansion for Overall and Low-Income Populations^a^

Characteristic	Estimated population, No.			Projected additional population without access (% difference)
	2008	2017	Change	
Total population without hospital <30-min drive				
Expansion states	10 107 000	10 528 000	421 000	NA
Nonexpansion states	7 456 000	8 206 000	750 000	NA
Nonexpansion states, projected^b^	NA	7 785 000	329 000	421 000 (–56.1)
Low-income population without hospital <30-min drive				
Expansion states	1 550 000	1 682 000	132 000	NA
Nonexpansion states	1 269 000	1 405 000	136 000	NA
Nonexpansion states, projected^b^	NA	1 357 000	88 000	48 000 (–35.3)
Total population without safety-net hospital <30-min drive				
Expansion states	60 633 000	64 409 000	3 776 000	NA
Nonexpansion states	51 520 000	57 723 000	6 203 000	NA
Nonexpansion states, projected^b^	NA	55 481 000	3 961 000	2 242 000 (–36.1)
Low-income population without safety-net hospital <30-min drive				
Expansion	8 064 000	8 869 000	805 000	NA
Nonexpansion	7 642 000	8 606 000	964 000	NA
Nonexpansion, projected^b^	NA	8 242 000	600 000	364 000 (–37.8)

Abbreviation: NA, not applicable.

^a^ Low-income population counts and locations were obtained using US Census data and Centers for Medicare and Medicaid Services Supplemental Security Income, and represented the population who reported income below the poverty line in the prior twelve months.^22,24,26^ State data obtained from public reporting as of December 2017. Expansion states were Alaska, Arkansas, Arizona, California, Colorado, Connecticut, District of Columbia, Hawaii, Iowa, Illinois, Indiana, Kentucky, Louisiana, Massachusetts, Maryland, Michigan, Minnesota, Montana, North Dakota, New Hampshire, New Jersey, New Mexico, Nevada, New York, Ohio, Oregon, Pennsylvania, Rhode Island, Vermont, Washington, and West Virginia. All other states were defined as nonexpansion.

^b^ Projected population in nonexpansion states that would have had access, if universal Medicaid expansion had occurred.

### Safety-Net Hospital Closures in States That Did and Did Not Expand Medicaid

Safety-net hospital closures occurred in half of states (26 of 51 [51%]), concentrated in the southeast United States (Figure 1). The majority of safety-net closures occurred in nonexpansion states (37 of 73 closures [51%]), with an additional 11 closures occurring in later-expanding states prior to their changes in Medicaid eligibility (cumulative 48 of 73 closures [65%]). Both expansion and nonexpansion states experienced net safety-net hospital closures in most years (Figure 2).

### Difference-in-Differences Analyses of Population Access to Safety-Net Hospitals

In the difference-in-differences analysis, states that did not expand Medicaid experienced an increase in the population without emergency access to a safety-net hospital (46.91% to 47.70% [0.79%]) compared with states that expanded Medicaid (33.94% to 33.07% [–0.87%]), for a difference-in-differences of 1.66% (95% CI, 1.64%-1.66%; *P* < .001; Table 1; Figure 3B; eTable 3 in the Supplement). States that did not expand Medicaid also experienced an increase in the lower income population without emergency access to a safety-net hospital (45.28% to 46.14% [0.86%]) compared with states that expanded Medicaid (33.00% to 32.23% [–0.77%]), for a difference-in-differences of 1.63% (95% CI, 1.61%-1.67%; *P* < .001; Table 1; eTable 3 and eFigure 2 in the Supplement). The projected population impact of not expanding Medicaid was a loss of emergency access to the nearest safety-net hospital for 2.2 million total persons and 364 000 low-income persons in 2017 for states that did not expand Medicaid (Table 2). These counts represent 36.1% and 37.8% declines in emergency access to safety-net hospitals in nonexpansion states attributable to Medicaid nonexpansion.

## Discussion

Timely access to an acute care hospital is a key determinant of improved clinical outcomes for conditions such as acute myocardial infarction,^2,3^ stroke,^4,6^ sepsis,^8,9^ and traumatic injuries.^10,11^ We found that states that did not expand Medicaid under the ACA experienced more acute care hospital closures, which was associated with a loss of timely access to care for an estimated 421 000 total persons, of whom 48 000 had low incomes. In states that did not expand Medicaid, an estimated additional 2.2 million persons overall and 364 thousand persons with low incomes lost timely access to safety-net acute care in association with safety-net hospital closures.

Our analysis contributes to the understanding of how state-level Medicaid expansion decisions impact public health for time-sensitive emergencies, both for the low-income and the overall population of the United States. While recent work has demonstrated an association between Medicaid expansion and hospital closures,^17,34^ summaries of closures alone cannot determine whether the shifting hospital landscape is associated with worsened, unchanged, or even potentially improved public health access for time-sensitive conditions.^35^ To answer this question, this drive-time analysis for all acute care hospitals in the United States before and after the ACA on an annual basis accounted for births, deaths, population migration, and rural-to-urban population changes to assess whether closures and new hospitals were associated with emergency access to acute care hospitals. Without such an analysis, it would not be possible to determine if emergency access was associated with changes in hospital service locations. This study found that changes in Medicaid eligibility at a state level may have negative repercussions for emergency access to acute care hospitals for persons at any income level.

Our analysis found a potential spillover effect from national health policy reform on changes in the local availability of services, with unanticipated and undesirable repercussions at patient, hospital, and regional levels. Policy makers should factor these results into decision-making when considering payment reforms and changes to health care entitlements like Medicaid. In addition, these results should spur policy makers to develop strategies to preserve population access independent of payment reform. Maryland and Pennsylvania, both Medicaid expansion states, have undertaken efforts to minimize financial uncertainty for smaller and rural hospitals through versions of capitated payment models.^36^ South Carolina, a nonexpansion state, has taken a different approach, creating incentives for hospitals to expand service delivery.^37^ It remains to be seen how well these programs maintain access to care and improve health care outcomes.

### Limitations

Our analysis has several limitations. We did not include hospital capacity in our models, and so true availability of services could have been overestimated or underestimated in some geographic regions. Similarly, while we limited inclusion to short-term acute care hospitals, we did not verify that all facilities had an operational emergency department or evaluate prehospital emergency services. We used the first full year of Medicaid expansion as the start of policy exposure, though financial strain from changes in payer-mix may not result in hospital closures within the first year. Indeed, inspection of the longitudinal changes curves suggests the impact of Medicaid expansion became more pronounced starting 2 years after expansion. Fourth, hospital closures in and of themselves are not necessarily undesirable. Closures of lower performing hospitals could result in improved health outcomes if there are nearby alternative hospitals. Indeed, studies evaluating closures alone have found inconsistent results,^35,38^ suggesting that other factors have a role in determining the public health impact of a closure.

## Conclusions

As of December 2017, 19 states had not expanded Medicaid under the ACA. This study shows that states that did not expand Medicaid under the ACA experienced worse emergency access to acute care hospitals compared with states that did. Unanticipated consequences of state decisions regarding Medicaid expansion include increased hospital closures and diminished emergency access to acute care hospitals. States choosing not to expand Medicaid should consider other ways to maintain critical public health infrastructure for acute care emergencies.

## Notes

Supplement. **eMethods.** **eTable 1.** Hospital and Population Characteristics in 2008 **eTable 2.** Hospital and Population Characteristics in 2017 **eTable 3.** Difference-in-Differences Analyses of Overall and Safety-Net Population Access **eTable 4.** Parallel Trends Assumption Testing **eTable 5.** Difference-in-Differences Analyses of Overall and Safety-Net Annual Hospital Counts **eTable 6.** Sensitivity Analyses: Difference-in-Differences Analyses of Overall and Safety-Net Hospital Counts and Population Access, Weighted by State Population **eFigure 1.** Lower Income Population Percentage Without Emergency Access to Any Short-Term Acute Care Hospital by Medicaid Expansion Status Under the Affordable Care Act **eFigure 2.** Lower Income Population Percentage Without Emergency Access to a Safety-Net Hospital by Medicaid Expansion Status Under the Affordable Care Act
