# Supplementary material for: Association Between State Medicaid Expansion and Emergency Access to Acute Care Hospitals in the United States
Source: JAMA Netw Open. 2020 Nov 16;3(11):e2025815. doi: 10.1001/jamanetworkopen.2020.25815 (PMC7670316; doi:10.1001/jamanetworkopen.2020.25815)
Supplement: Supplement. — eMethods. eTable 1. Hospital and Population Characteristics in 2008 eTable 2. Hospital and Population Characteristics in 2017 eTable 3. Difference-in-Differences Analyses of Overall and Safety-Net Population Access eTable 4. Parallel Trends Assumption Testing eTable 5. Difference-in-Differences Analyses of Overall and Safety-Net Annual Hospital Counts eTable 6. Sensitivity Analyses: Difference-in-Differences Analyses of Overall and Safety-Net Hospital Counts and Population Access, Weighted by State Population eFigure 1. Lower Income Population Percentage Without Emergency Access to Any Short-Term Acute Care Hospital by Medicaid Expansion Status Under the Affordable Care Act eFigure 2. Lower Income Population Percentage Without Emergency Access to a Safety-Net Hospital by Medicaid Expansion Status Under the Affordable Care Act [file jamanetwopen-e2025815-s001.pdf]

## Supplemental Online Content

Wallace DJ, Donohue JM, Angus DC, et al. Association between state Medicaid expansion and emergency access to acute care hospitals in the United States. *JAMA Netw Open*. 2020;3(11):e2025815. doi:10.1001/jamanetworkopen.2020.25815

**eMethods.**
**eTable 1.** Hospital and Population Characteristics in 2008
**eTable 2.** Hospital and Population Characteristics in 2017
**eTable 3.** Difference-in-Differences Analyses of Overall and Safety-Net Population Access
**eTable 4.** Parallel Trends Assumption Testing
**eTable 5.** Difference-in-Differences Analyses of Overall and Safety-Net Annual Hospital Counts
**eTable 6.** Sensitivity Analyses: Difference-in-Differences Analyses of Overall and Safety-Net Hospital Counts and Population Access, Weighted by State Population
**eFigure 1.** Lower Income Population Percentage Without Emergency Access to Any Short-term Acute Care Hospital by Medicaid Expansion Status Under the Affordable Care Act
**eFigure 2.** Lower Income Population Percentage Without Emergency Access to a Safety-net Hospital by Medicaid Expansion Status Under the Affordable Care Act

This supplemental material has been provided by the authors to give readers additional information about their work.

© 2020 Wallace DJ et al. *JAMA Network Open.*

## eMethods

### Parallel trends assumption evaluation

For the pre-expansion period only, we tested the parallel trends assumption using the following model:

$$Y_{it} = \eta_0 + \eta_1 Treatment_i + \tau_0 Time_t + \tau_1 (Treatment_i * Time_t) + \epsilon_{it}$$

where $Y_{it}$ was the outcome of interest for state $i$ in relative year $t$, $Treatment_i$ is an indicator equal to 1 for non-expansion states, $Time_t$ was a continuous time variable (where year 0 is the final pre-expansion year), and $\epsilon_{it}$ was a state-year level error term. We tested the null hypothesis that the time by treatment interaction term ($\tau_1$) is zero. If this hypothesis was not rejected, we would conclude that the parallel trends assumption was met. For the full time period, the difference-in-differences model was:

$$Y_{it} = \eta_0 + \eta_1 Treatment_i + \varphi_0 Post_t + \beta (Treatment_i * Post_t) + \epsilon_{it}$$

where $Post_t$ was an indicator equal to 1 in the post period and $\epsilon_{it}$ was a state-time period level error term. We tested the null hypothesis that the post by treatment interaction term ($\beta$) was zero. If this hypothesis was not rejected, we would conclude that Medicaid non-expansion did not have a significant effect on the outcome of interest. If this hypothesis was rejected, we would conclude that non-expansion had an impact.

We used cluster-robust standard errors, clustered at the state level.

### Difference-in-differences analyses of changes in numbers of active hospitals by year, between states that expanded Medicaid and states that did not

We applied a difference-in-differences analysis for our longitudinal evaluation of annual changes in hospital counts between expansion and non-expansion states. We summarized changes in hospital counts at a state-level in each year of the analysis, using the change in count as the outcome variable. We used a linear regression model that included terms for year

© 2020 Wallace DJ et al. *JAMA Network Open.*

relative to Medicaid expansion (which was 2014 for most expansion states), year, non-expansion indicator variable, and an interaction term between relative year and expansion status.

We performed a planned sensitivity analysis of adding a state-level population weight term to the difference-in-differences model, for dependent variables overall hospital count and safety-net hospital count. We performed this sensitivity analysis to determine if our main analysis findings are robust to controls for the size of the population.

### Rural population count and percentage estimations

We calculated geodemographic population counts and percentages living in rural areas using data from the US Census Bureau American Community Survey[24] and TIGER shapefiles. We merged annual US Census population estimates with US Census 2010 ZCTA code shapefiles, creating a population map for each year. We used a 2010 ZCTA code shapefile for 2008 calculations, rather than a US Census 2000 ZIP code shapefile, to avoid differences between year 2000 and year 2010 ZIP code apportionment methodology. We then summarized the populations of ZIP codes that intersected Census Urban Areas regions. We used Urban Area designations as these are regions identified by the Census Bureau as having dense populations of 50,000 persons or more.

## eResults

### Parallel trends assumption evaluation for acute care hospitals

The interaction term F-statistic (1, 263) was 2.79, with a probability of that result or greater by chance of 0.10 (eTable 4). We therefore did not reject the null hypothesis and proceeded to use a difference-in-differences analytic approach.

### Parallel trends assumption evaluation for safety-net hospitals

© 2020 Wallace DJ et al. *JAMA Network Open.*

The interaction term F-statistic (1, 263) was 4.02, with a probability of that result or greater by chance of 0.05. Of note, though this result was close to our prespecified level of statistical significance, visual inspection of the change in safety-net hospital trend reveals an attenuation of hospital losses in expansion states and an increase in safety-net loses in non-expansion states, compared to relatively consistent loses in the pre-periods. We therefore did not reject the null hypothesis and proceeded to use a difference-in-differences analytic approach.

**Sensitivity analyses: difference-in-differences analyses of changes in numbers of active hospitals by year, between states that expanded Medicaid and states that did not, with state-population weighting**

The results of the sensitivity analyses that included a state-level population weight term were identical to the main analysis (eTable 6). The interaction terms in both the general hospital model and the safety-net hospital model both had negative coefficients and both were statistically significant, indicating that there was a lower relative count of hospitals after expansion in states that did not expand Medicaid, compared to those that did – while controlling for the state-level population.

© 2020 Wallace DJ et al. *JAMA Network Open.*

| eTable 1. Hospital and Population Characteristics in 2008 | | | |
|---|---|---|---|
| | Medicaid Expansion[b] | Medicaid Non-Expansion[b] | Total |
| **Hospitals,[a] n (%)** | | | |
| Status | | | |
| Active | 2,587 (99.7) | 2,014 (99.3) | 4,601 (99.5) |
| New | 7 (0.3) | 6 (0.3) | 13 (0.3) |
| Closed | 7 (0.3) | 14 (0.7) | 21 (0.5) |
| Hospital beds | | | |
| Less than 249 | 2,169 (83.8) | 1,744 (86.6) | 3,913 (85.0) |
| 250-499 | 364 (14.1) | 221 (11.0) | 585 (12.7) |
| More than 499 | 54 (2.1) | 49 (2.4) | 103 (2.2) |
| ICU beds | | | |
| 0-5 | 926 (35.8) | 928 (46.1) | 1,854 (40.3) |
| 6-19 | 866 (33.5) | 598 (29.7) | 1,464 (31.8) |
| More than 19 | 795 (30.7) | 488 (24.2) | 1,283 (27.9) |
| Teaching status[c] | | | |
| Non-teaching | 1,712 (66.2) | 1,614 (80.1) | 3,326 (72.3) |
| Minor teaching | 595 (23.0) | 329 (16.3) | 924 (20.1) |
| Major teaching | 280 (10.8) | 71 (3.5) | 351 (7.6) |
| Financial status | | | |
| Non-profit | 1,791 (69.2) | 899 (44.6) | 2,690 (58.5) |
| Government | 435 (16.8) | 584 (29.0) | 1,019 (22.1) |
| For-profit | 361 (14.0) | 531 (26.4) | 892 (19.4) |
| Safety-net status[d] | 626 (24.2) | 492 (24.4) | 1,118 (24.3) |
| **Populations, n (%)** | | | |
| Total population[e] | 181,575,200 | 110,291,344 | 291,866,544 |
| Total population living more than 30-minutes from nearest hospital | 10,106,544 (5.6) | 7,456,472 (6.8) | 17,563,016 (6.0) |
| Total population living more than 30-minutes from nearest safety-net hospital | 60,632,696 (33.4) | 51,519,808 (46.7) | 112,152,504 (38.4) |
| Total population living a rural location[e] | 37,309,936 (20.5) | 26,343,942 (23.9) | 63,653,878 (21.8) |
| Low-income population[e] | 24,812,190 | 16,937,140 | 41,749,330 |
| Low-income population living more than 30-minutes from nearest hospital | 1,550,350 (6.2) | 1,268,976 (7.5) | 2,819,326 (6.8) |
| Low-income population living more than 30-minutes from nearest safety-net hospital | 8,063,527 (32.5) | 7,641,968 (45.1) | 15,705,495 (37.6) |
| Low-income population living a rural location[e] | 5,358,408 (21.6) | 4,898,821 (28.9) | 10,257,229 (24.6) |

Abbreviation: ICU, intensive care unit.

[a] Obtained from Centers for Medicare and Medicaid Services Healthcare Cost Report Information System annual reports for 2008.[40]

[b] Obtained from public reporting as of December 2017. Expansion states were Alaska, Arkansas, Arizona, California, Colorado, Connecticut, District of Columbia, Hawaii, Iowa, Illinois, Indiana, Kentucky, Louisiana, Massachusetts, Maryland, Michigan, Minnesota, Montana, North Dakota, New Hampshire, New Jersey, New Mexico, Nevada, New York, Ohio, Oregon, Pennsylvania, Rhode Island, Vermont, Washington, and West Virginia. All other states were defined as non-expansion.

[c] Defined using the ratio of residents to hospital beds. Major teaching were hospitals with a resident-to-bed ratio > 0.25, minor teaching were hospitals with a resident-to-bed ratio > 0 but ≤ 0.25, and non-teaching were hospitals with a resident-to-bed ratio of zero.[41]

[d] Obtained from Centers for Medicare and Medicaid Services Supplemental Security Income files.[22,27]

[e] Obtained from the United States Census Bureau.[24]

© 2020 Wallace DJ et al. *JAMA Network Open.*

| eTable 2. Hospital and Population Characteristics in 2017 | | | |
|---|---|---|---|
| | **Medicaid Expansion**[b] | **Medicaid Non-Expansion**[b] | **Total** |
| **Hospitals,[a] n (%)** | | | |
| Status | | | |
| Active | 2,542 (99.9) | 1,986 (99.2) | 4,528 (99.6) |
| New | 6 (0.2) | 6 (0.3) | 12 (0.3) |
| Closed | 3 (0.1) | 16 (0.8) | 19 (0.4) |
| Hospital beds | | | |
| Less than 249 | 2,146 (84.4) | 1,715 (86.4) | 3,861 (85.3) |
| 250-499 | 330 (13.0) | 218 (11.0) | 548 (12.1) |
| More than 499 | 66 (2.6) | 53 (2.7) | 119 (2.6) |
| ICU beds | | | |
| 0-5 | 937 (36.9) | 897 (45.2) | 1,834 (40.5) |
| 6-19 | 769 (30.3) | 556 (28.0) | 1,325 (29.3) |
| More than 19 | 836 (32.9) | 533 (26.8) | 1,369 (30.2) |
| Teaching status[c] | | | |
| Non-teaching | 1,684 (66.2) | 1,589 (80.0) | 3,273 (72.3) |
| Minor teaching | 532 (20.9) | 303 (15.3) | 835 (18.4) |
| Major teaching | 326 (12.8) | 94 (4.7) | 420 (9.3) |
| Financial status | | | |
| Non-profit | 1,771 (69.7) | 909 (45.9) | 2,680 (59.2) |
| Government | 428 (16.8) | 563 (28.3) | 991 (21.9) |
| For-profit | 343 (13.5) | 514 (25.9) | 857 (18.9) |
| Safety-net status[d], n (%) | 590 (23.2) | 455 (22.9) | 1,045 (23.1) |
| **Populations, n (%)** | | | |
| Total population[e] | 192,536,768 | 120,507,096 | 313,043,864 |
| Total population living more than 30-minutes from nearest hospital | 10,527,664 (5.5) | 8,206,440 (6.8) | 18,734,104 (6.0) |
| Total population living more than 30-minutes from nearest safety-net hospital | 64,409,144 (33.5) | 57,723,120 (47.9) | 122,132,264 (39.0) |
| Total population living a rural location[e] | 38,698,069 (20.1) | 26,908,911 (22.3) | 65,606,980 (21.0) |
| Low-income population[e] | 27,133,328 | 18,516,470 | 45,649,798 |
| Low-income population living more than 30-minutes from nearest hospital | 1,682,242 (6.2) | 1,404,830 (7.6) | 3,087,072 (6.8) |
| Low-income population living more than 30-minutes from nearest safety-net hospital | 8,868,652 (32.7) | 8,605,936 (46.5) | 17,474,588 (38.3) |
| Low-income population living a rural location[e] | 5,664,947 (20.9) | 5,024,546 (27.1) | 10,689,493 (23.4) |

Abbreviation: ICU, intensive care unit.

[a] Obtained from Centers for Medicare and Medicaid Services Healthcare Cost Report Information System annual reports for 2017.[40]

[b] Obtained from public reporting as of December 2017. Expansion states were Alaska, Arkansas, Arizona, California, Colorado, Connecticut, District of Columbia, Hawaii, Iowa, Illinois, Indiana, Kentucky, Louisiana, Massachusetts, Maryland, Michigan, Minnesota, Montana, North Dakota, New Hampshire, New Jersey, New Mexico, Nevada, New York, Ohio, Oregon, Pennsylvania, Rhode Island, Vermont, Washington, and West Virginia. All other states were defined as non-expansion.

[c] Defined using the ratio of residents to hospital beds. Major teaching were hospitals with a resident-to-bed ratio > 0.25, minor teaching were hospitals with a resident-to-bed ratio > 0 but ≤ 0.25, and non-teaching were hospitals with a resident-to-bed ratio of zero.[41]

[d] Obtained from Centers for Medicare and Medicaid Services Supplemental Security Income files.[22,27]

[e] Obtained from the United States Census Bureau.[24]

© 2020 Wallace DJ et al. *JAMA Network Open.*

| eTable 3. Difference-in-Differences Analyses of Overall[a] and Safety-Net[a,b] Population Access | | | |
|---|---|---|---|
| **Overall Population Access to Any Hospital** | **Access Population Percentage** | **95% Confidence Interval (CI)** | **P-value** |
| Baseline | 94.35 | 94.35 to 94.35 | < 0.01 |
| Non-Expansion Status[c] | -1.11 | -1.11to -1.11 | < 0.01 |
| Post-Expansion Status[d] | 0.30 | 0.29 to 0.30 | < 0.01 |
| Interaction | -0.33 | -0.32 to -0.33 | < 0.01 |
| **Low-Income Population Access to Any Hospital** | | | |
| Baseline | 93.72 | 93.71 to 93.73 | < 0.01 |
| Non-Expansion Status[c] | -1.15 | -1.14 to -1.16 | < 0.01 |
| Post-Expansion Status[d] | 0.17 | 0.16 to 0.18 | < 0.01 |
| Interaction | -0.13 | -0.11 to -0.14 | < 0.01 |
| **Overall Population Access to a Safety-Net Hospital** | | | |
| Baseline | 66.07 | 66.07 to 66.08 | < 0.01 |
| Non-Expansion Status[c] | -12.99 | -12.98 to -12.99 | < 0.01 |
| Post-Expansion Status[d] | 0.83 | 0.82 to 0.83 | < 0.01 |
| Interaction | -1.61 | -1.60 to -1.61 | < 0.01 |
| **Low-Income Population Access to a Safety-Net Hospital** | | | |
| Baseline | 67.01 | 67.00 to 67.02 | < 0.01 |
| Non-Expansion Status[c] | -12.29 | -12.27 to -12.31 | < 0.01 |
| Post-Expansion Status[d] | 0.74 | 0.72 to 0.76 | < 0.01 |
| Interaction | -1.61 | -1.58 to -1.64 | < 0.01 |

[a] Obtained from Centers for Medicare and Medicaid Services Healthcare Cost Report Information System annual reports.[40]

[b] Obtained from Centers for Medicare and Medicaid Services Supplemental Security Income files.[22,27]

[c] Obtained from public reporting as of December 2017. Expansion states were Alaska, Arkansas, Arizona, California, Colorado, Connecticut, District of Columbia, Hawaii, Iowa, Illinois, Indiana, Kentucky, Louisiana, Massachusetts, Maryland, Michigan, Minnesota, Montana, North Dakota, New Hampshire, New Jersey, New Mexico, Nevada, New York, Ohio, Oregon, Pennsylvania, Rhode Island, Vermont, Washington, and West Virginia. All other states were defined as non-expansion.

[d] Defined by the first full calendar year of Medicaid expansion in expansion states and 2014 in non-expansion states.

© 2020 Wallace DJ et al. *JAMA Network Open.*

| eTable 4. Parallel Trends Assumption Testing | | | | |
|---|---|---|---|---|
| **Variable** | **Coefficient** | **Robust Standard Error** | **p-value** | **95% Confidence Interval** |
| Non-expansion[a] | -0.0013 | 0.0023 | 0.57 | (-0.0059 – 0.0033) |
| Relative year[b] | 0.0007 | 0.0005 | 0.14 | (-0.0002 – 0.0017) |
| Interaction | -0.0016 | 0.0010 | 0.10 | (-0.0035 – 0.0003) |
| Constant | 0.0001 | 0.0011 | 0.91 | (-0.0020 – 0.0022) |

[a] Obtained from public reporting as of December 2017. Expansion states were Alaska, Arkansas, Arizona, California, Colorado, Connecticut, District of Columbia, Hawaii, Iowa, Illinois, Indiana, Kentucky, Louisiana, Massachusetts, Maryland, Michigan, Minnesota, Montana, North Dakota, New Hampshire, New Jersey, New Mexico, Nevada, New York, Ohio, Oregon, Pennsylvania, Rhode Island, Vermont, Washington, and West Virginia. All other states were defined as non-expansion.

[b] Defined as number of years relative to the first full calendar year of Medicaid expansion, in expansion states, and 2014 in non-expansion states.

© 2020 Wallace DJ et al. *JAMA Network Open.*

| eTable 5. Difference-in-Differences Analyses of Overall[a] and Safety-Net[a,b] Annual Hospital Counts | | | |
|---|---|---|---|
| **Overall Hospital Counts[a]** | **Average Annual Percent Change** | **Percent Change 95% Confidence Interval (CI)** | **P-value** |
| Baseline | -0.15 | -0.29 to -0.01 | 0.03 |
| Non-Expansion Status[c] | 0.21[e] | -0.05 to 0.46 | 0.11 |
| Post-Expansion Status[d] | 0.14 | -0.06 to 0.33 | 0.17 |
| Interaction | -0.51[f] | -0.88 to -0.13 | 0.01 |
| **Safety-Net Hospital Counts[a,b]** | | | |
| Baseline | -0.48 | -0.79 to -0.18 | < 0.01 |
| Non-Expansion Status[c] | 0.10[g] | -0.35 to 0.54 | 0.66 |
| Post-Expansion Status[d] | 0.17 | -0.23 to 0.57 | 0.39 |
| Interaction | -1.02[h] | -1.79 to -0.26 | 0.01 |

[a] Obtained from Centers for Medicare and Medicaid Services Healthcare Cost Report Information System annual reports.[40]

[b] Obtained from Centers for Medicare and Medicaid Services Supplemental Security Income files [22,27].

[c] Obtained from public reporting as of December 2017. Expansion states were Alaska, Arkansas, Arizona, California, Colorado, Connecticut, District of Columbia, Hawaii, Iowa, Illinois, Indiana, Kentucky, Louisiana, Massachusetts, Maryland, Michigan, Minnesota, Montana, North Dakota, New Hampshire, New Jersey, New Mexico, Nevada, New York, Ohio, Oregon, Pennsylvania, Rhode Island, Vermont, Washington, and West Virginia. All other states were defined as non-expansion.

[d] Defined by the first full calendar year of Medicaid expansion in expansion states and 2014 in non-expansion states.

[e] Before Medicaid expansion, there was no difference in average annual change in hospital numbers between expansion and non-expansion states (percent change +0.21%; 95%CI:-0.05 to 0.46; p=0.11 ).

[f] After Medicaid expansion, average annual change in hospital numbers differed between expansion and non-expansion states by -0.51% (95%CI: -0.88 to -0.13; p=0.01), such that non-expansion states experienced an average change in hospital numbers of -0.31% (-0.15 plus +0.21 plus +0.14 plus -0.51), compared to expansion states that experienced an average annual change in hospital numbers of 0.01% (-0.15 plus +0.14).

[g] Before Medicaid expansion, there was no difference in average annual change in safety-net hospital numbers between expansion and non-expansion states (percent change 0.10%; 95%CI: -0.35 to 0.55; p=0.66).

[h] After Medicaid expansion, average annual change in safety-net hospital numbers differed between expansion and non-expansion states by -1.02% (95%CI: -1.79 to -0.26%; p=0.01), such that non-expansion states experienced an average annual decline in safety-net hospital numbers of -1.23% (-0.48 plus 0.01 plus +0.17 plus -1.02), compared to expansion states that experienced an average annual change in safety-net hospital numbers of -0.31% (-0.48 plus 0.17%).

| eTable 6. Sensitivity Analyses: Difference-in-Differences Analyses of Overall[a] and Safety-Net[a,b] Hospital Counts and Population Access[c], Weighted by State Population[c] | | | |
|---|---|---|---|
| **Overall Hospital Counts** | **Average Annual Percent Change** | **Annual Percent Change 95% Confidence Interval (CI)** | **P-value** |
| Baseline | -0.31 | -0.50 to -0.13 | <0.01 |
| Non-Expansion Status[d] | +0.40 | 0.12 to +0.69 | 0.01 |
| Post-Medicaid Expansion Status[e] | +0.23 | 0.01 to +0.46 | 0.05 |
| Interaction | -0.69 | -1.07 to -0.30 | <0.01 |
| **Safety-Net Hospital Counts** | | | |
| Baseline | -0.90 | -1.30 to -0.51 | <0.01 |
| Non-Expansion Status[d] | 0.48 | -0.01 to 0.97 | 0.06 |
| Post-Medicaid Expansion Status[e] | 0.47 | 0.01 to 0.94 | 0.05 |
| Interaction | -1.33 | -2.10 to -0.57 | <0.01 |

[a] Obtained from Centers for Medicare and Medicaid Services Healthcare Cost Report Information System annual reports.[40]
[b] Obtained from Centers for Medicare and Medicaid Services Supplemental Security Income files [22,27].
[c] Obtained from the United States Census Bureau.[24]
[d] Obtained from public reporting as of December 2017. Expansion states were Alaska, Arkansas, Arizona, California, Colorado, Connecticut, District of Columbia, Hawaii, Iowa, Illinois, Indiana, Kentucky, Louisiana, Massachusetts, Maryland, Michigan, Minnesota, Montana, North Dakota, New Hampshire, New Jersey, New Mexico, Nevada, New York, Ohio, Oregon, Pennsylvania, Rhode Island, Vermont, Washington, and West Virginia. All other states were defined as non-expansion.
[e] Defined by the first full calendar year of Medicaid expansion in expansion states and 2014 in non-expansion states.

© 2020 Wallace DJ et al. *JAMA Network Open.*

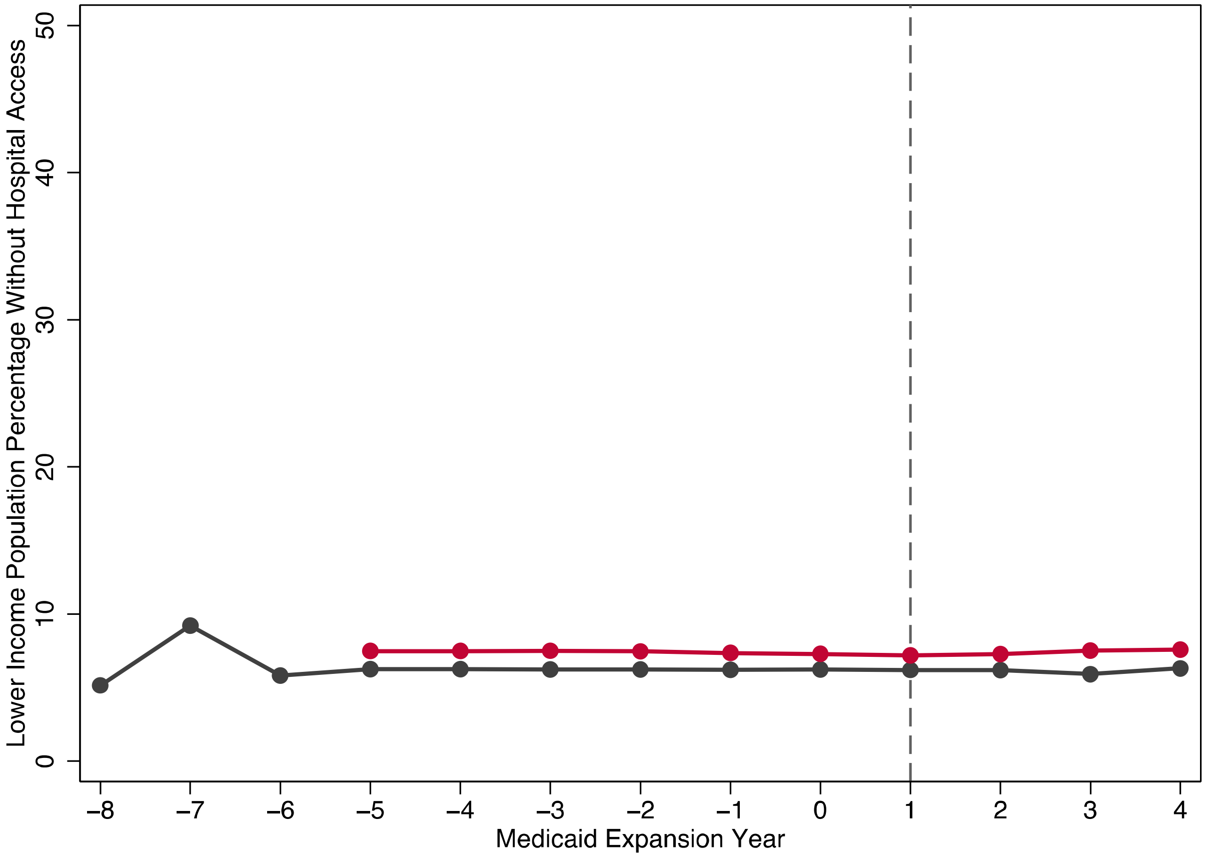


**eFigure 1. Lower income population percentage without emergency access to any short-term acute care hospital by Medicaid expansion status under the Affordable Care Act.** Thirty-two states expanded Medicaid eligibility requirements between 2014 and 2017, and nineteen states did not. The **grey dashed line** references the first year of Medicaid expansion among states that expanded Medicaid and references the year 2014 for states that did not. The **solid red line** shows the percentage of the lower income population without emergency access to any acute care hospital in states that did not expand Medicaid. The **solid gray line** shows the percentage of the lower income population without emergency access to any acute care hospital in states that expanded Medicaid.

© 2020 Wallace DJ et al. *JAMA Network Open.*

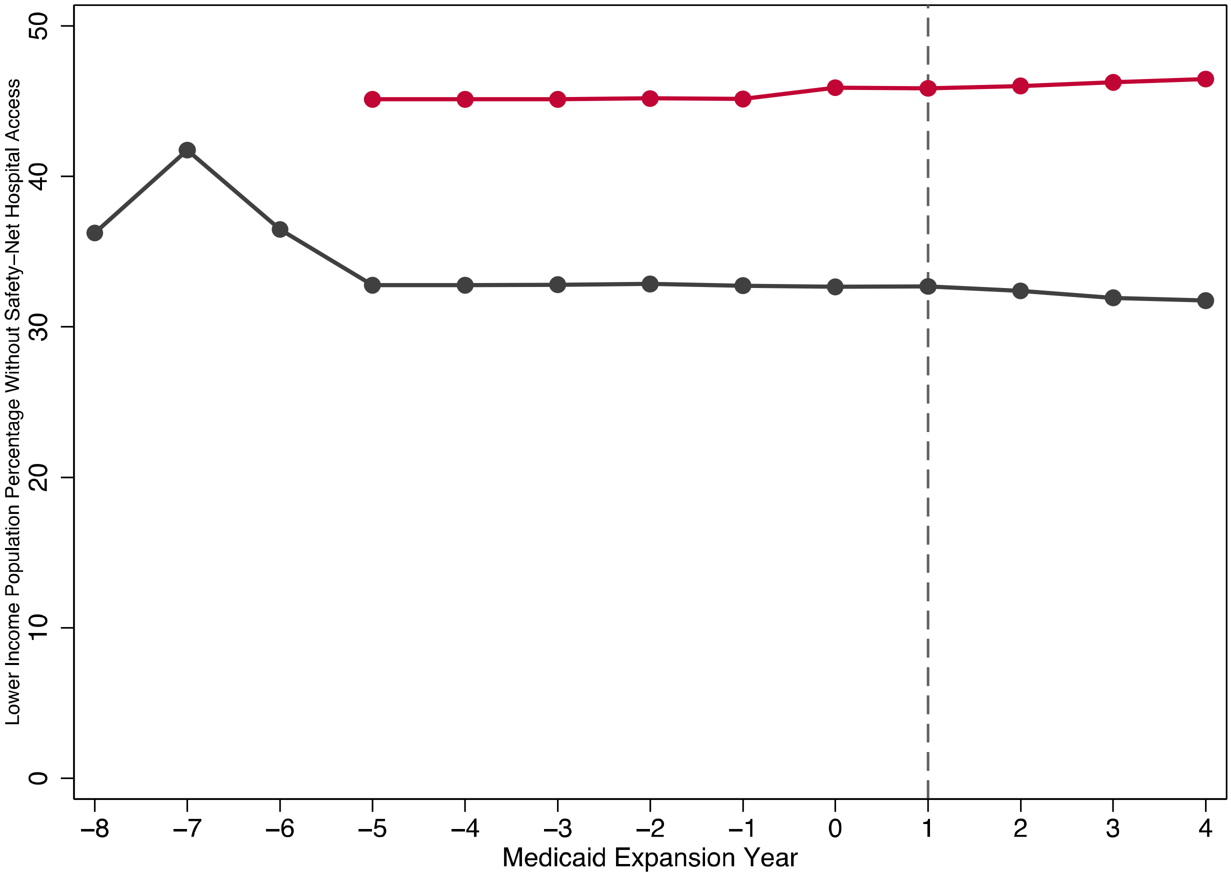


**eFigure 2. Lower income population percentage without emergency access to a safety-net hospital by Medicaid expansion status under the Affordable Care Act.** Thirty-two states expanded Medicaid eligibility requirements between 2014 and 2017, and nineteen states did not. The **grey dashed line** references the first year of Medicaid expansion among states that expanded Medicaid and references the year 2014 for states that did not. The **solid red line** shows the percentage of the lower income population without emergency access to a safety-net hospital in states that did not expand Medicaid. The **solid gray line** shows the percentage of the lower income population without emergency access to a safety-net hospital in states that expanded Medicaid.

© 2020 Wallace DJ et al. *JAMA Network Open.*
